# *MDM2* Promoter SNP344T>A (rs1196333) Status Does Not Affect Cancer Risk

**DOI:** 10.1371/journal.pone.0036263

**Published:** 2012-04-30

**Authors:** Stian Knappskog, Liv B. Gansmo, Pål Romundstad, Merete Bjørnslett, Jone Trovik, Jan Sommerfelt-Pettersen, Erik Løkkevik, for the Norwegian Breast Cancer Group trial NBCG VI, Rob A. E. M. Tollenaar, Caroline Seynaeve, Peter Devilee, Helga B. Salvesen, Anne Dørum, Kristian Hveem, Lars Vatten, Per E. Lønning

**Affiliations:** 1 Section of Oncology, Institute of Medicine, University of Bergen, Bergen, Norway; 2 Department of Oncology, Haukeland University Hospital, Bergen, Norway; 3 Department of Public Health, Faculty of Medicine, Norwegian University of Science and Technology, Trondheim, Norway; 4 Department of Medical Genetics, Oslo University Hospital Radiumhospitalet, Oslo, Norway; 5 Faculty Division, The Norwegian Radium Hospital, University of Oslo, Oslo, Norway; 6 Department of Obstetrics and Gynecology, Haukeland University Hospital, Bergen, Norway; 7 Institute of Clinical Medicine, University of Bergen, Norway; 8 Director Naval Medicine, Royal Norwegian Navy, Bergen, Norway; 9 Division of Surgery and Cancer Medicine, Department of Oncology, Oslo University Hospital, Oslo, Norway; 10 Department of Surgery, Leiden University Medical Center, Leiden, The Netherlands; 11 Department of Medical Oncology, Family Cancer Clinic, Erasmus MC-Daniel den Hoed Cancer Center Rotterdam, Rotterdam, The Netherlands; 12 Department of Human Genetics, Leiden University Medical Center, Leiden, The Netherlands; 13 Department of Pathology, Leiden University Medical Center, Leiden, The Netherlands; 14 Department of Gynecological Oncology, Oslo University Hospital Radiumhospitalet, Oslo, Norway; University of Saarland, Germany

## Abstract

The *MDM2* proto-oncogene plays a key role in central cellular processes like growth control and apoptosis, and the gene locus is frequently amplified in sarcomas. Two polymorphisms located in the MDM2 promoter P2 have been shown to affect cancer risk. One of these polymorphisms (SNP309T>G; rs2279744) facilitates Sp1 transcription factor binding to the promoter and is associated with increased cancer risk. In contrast, SNP285G>C (rs117039649), located 24 bp upstream of rs2279744, and in complete linkage disequilibrium with the SNP309G allele, reduces Sp1 recruitment and lowers cancer risk. Thus, fine tuning of MDM2 expression has proven to be of significant importance with respect to tumorigenesis. We assessed the potential functional effects of a third *MDM2* promoter P2 polymorphism (SNP344T>A; rs1196333) located on the SNP309T allele. While *in silico* analyses indicated SNP344A to modulate TFAP2A, SPIB and AP1 transcription factor binding, we found no effect of SNP344 status on MDM2 expression levels. Assessing the frequency of SNP344A in healthy Caucasians (n = 2,954) and patients suffering from ovarian (n = 1,927), breast (n = 1,271), endometrial (n = 895) or prostatic cancer (n = 641), we detected no significant difference in the distribution of this polymorphism between any of these cancer forms and healthy controls (6.1% in healthy controls, and 4.9%, 5.0%, 5.4% and 7.2% in the cancer groups, respectively). In conclusion, our findings provide no evidence indicating that SNP344A may affect MDM2 transcription or cancer risk.

## Introduction

The Mouse Double Minute 2 homolog (MDM2) is a key regulator of p53 as well as retinoblastoma protein function [1], [2], [3]. Thus, elevated MDM2 protein levels due to *MDM2* gene amplification or other mechanisms have been regarded as an alternative to *TP53* mutations diminishing p53 function in many human cancers [2], [4], [5], [6], [7].

In 2004 the group of A. Levine discovered a polymorphism SNP309T>G (rs2279744) in the *MDM2* intronic P2 promoter [8]. SNP309G enhances *MDM2* expression levels by increasing Sp1 transcription factor binding and was subsequently shown to be associated with increased risk and an early age at diagnosis of several malignancies [8], [9], [10].

While subsequent studies have confirmed an association between SNP309G and the risk of multiple cancer forms, the effect of this SNP seems to differ between ethnic groups: Thus, while most studies performed in Asian or Ashkenazi Jewish populations reports the SNP309G variant to enhance cancer risk many studies conducted in Caucasian populations have failed to reproduce a similar effect [11], [12].

Recently, we reported a second polymorphism, SNP285G>C (rs117039649), located 24 base-pairs from SNP309 in the *MDM2* P2 promoter. The SNP285C variant allele is observed among Caucasians only, in whom it forms a distinct SNP285C/309G haplotype accounting for about 12% of the SNP309G alleles [13]. SNP285C antagonizes the effect of SNP309G by reducing Sp1 transcription factor binding strength to the *MDM2* promoter and is associated with a reduced risk for breast, ovarian and endometrial cancers [13], [14].

Taken together, the data from the studies on SNP309 and SNP285 strongly indicate fine tuning of *MDM2* P2 promoter activity to be of importance for cancer risk. It is therefore of interest to search for additional variants in the *MDM2* promoter that may contribute to altered cancer risk.

SNP344T>A (rs1196333), located 35 base pairs downstream of SNP309, was initially identified by Bond et al [8] in 4 out of 50 healthy individuals. Here, we present the first report assessing the impact of SNP344 status on MDM2 expression as well as cancer risk in large populations. Thus, we have examined its impact on the risk of ovarian, breast, endometrial and prostate cancer, and studied the same patient cohorts for which the risk profiles linked to SNP309T>G and SNP285G>C have previously been analysed in detail [13], [14].

## Materials and Methods

### *MDM2* promoter SNP344 status screening

A region of the *MDM2* promoter P2 containing SNP344 (as well as SNP285 and SNP309) was previously amplified by PCR, sequenced and analysed for SNP285 and SNP309 status [13], [14]. Here, these sequence traces were analysed for SNP344 status.

### *In silico* predictions

Predictions of potential transcription binding sites in the *MDM2* promoter affected by SNP344 status were performed using the JASPAR database at http://jaspar.genereg.net [15]. Input sequences for the predictions were *tgcctgtcgggtca* for the SNP344T-allele and *tgcctgacgggtca* for the SNP344A-allele. Profile score threshold were set to 80% (default settings).

### MDM2 expression analysis

Total RNA was extracted from white blood cells drawn from 215 young males as part of a routine test during conscription in the Navy [13] using Trizol reagent (Life technologies) according to the manufacture's protocol, and dissolved in DEPC treated ddH_2_O.

Single strand cDNA synthesis was performed using 500 ng total RNA, oligo-dT- and random hexamer primers (Sigma) with Transcriptor Reverse Transcriptase (Roche) in accordance with the manufacturer's instructions. After RT-PCR the cDNA was diluted 1∶10 in ddH_2_O.

Quantitative PCRs for total MDM2 expression levels, MDM2 promoter 2 specific expression and RPLP2 (internal reference) were carried out using Hydrolysis probes (TIB MOLBIOL) on a Ligthcycler 480 instrument (Roche). The following primers were used: MDM2_F; aacatgtacctactgatggtgc, MDM2_R; cagggtctcttgttccgaagc, MDM2_TM; 6FAM-aaccacctcacagattcc-BBQ, MDM2P2_S; gcgattggagggtagacctgt, MDM2P2_R: ggtattgcacatttgcctggat, MDM2P2_TM; 6FAM-agtggcgtgcgtccgtgcc-BBQ, RPLP2_F; gaccggctcaacaaggttat, RPLP2_A; ccccaccagcaggtacac and RPLP2_TM; 6FAM-agctgaatggaaaaaacattgaagacgtc-BBQ. Amplifications were performed in a reaction volume of 10 µl using the LigthCycler® 480 Probes Master kit (Roche) with 0,5 µM of forward and reverse primer, 0,125 µM of each hydrolysis probe and 3 µl cDNA. The thermocycling conditions were: 5 min initial denaturation at 95°C, before 45 cycles at 95°C for 10 s and at 55°C for 20 s, and a final cooling step at 40°C for 10 s. Relative MDM2 mRNA concentrations were calculated based on in-run standard curves and normalization to RPLP2 mRNA levels in the same samples. Water was included in each run as negative control and all analyses were performed in triplicate runs.

### Healthy Caucasian controls

The distribution of *MDM2* SNP344 among cancer patients was compared to 2,954 Norwegian healthy controls. The controls have been described in detail previously [13], [14].

### African American individuals

DNA from African American individuals (n = 50) was purchased from Coriell Institute for Medical Research (Cat # HD50AA).

### Cancer patients

Ovarian (n = 1,927), breast (n = 1,271), endometrial (n = 895) and prostate cancer patients (n = 641) were from patient cohorts that were previously analysed for *MDM2* SNP285 and SNP309 [13], [14].

### Ethics Considerations

Collection and use of samples from cancer patients and healthy controls, as well as controls for expression analyses, was approved by the Regional Ethical Committees of Western Norway (Haukeland University Hospital), Central Norway (Norwegian University of Science and Technology), and South-Eastern Norway (Oslo University Hospital Radiumhospitalet; Norwegian samples), and The Medical Ethical Committees of the Leiden University Medical Center, Leiden, The Netherlands, and the Erasmus MC-Daniel den Hoed Cancer Center Rotterdam, The Netherlands (Dutch samples). All participants gave written informed consent.

### Statistical analysis

Expression levels of MDM2 between individuals with the different genotypes of SNP344 were compared using the Mann-Whitney rank test. Among individuals for whom MDM2 expression was analysed (n = 215), one harboured the SNP344AA genotype. For statistical calculations, this individual was included in the SNP344TA group and compared to the SNP344TT group.

Potential differences in the distribution of SNP344 between cancer patients and healthy controls as well as between subgroups of each cancer form were assessed by Odds Ratios (OR) and by Fischer exact test. ORs are given with 95% confidence intervals (CI).

Potential differences in age at onset of disease between the patients were assessed by Kruskal-Wallis rank tests.

Survival was assessed by Kaplan–Meier analyses where the different patients groups were compared using the log rank test; deaths for reasons other than breast cancer were censored.

All p-values are two-sided, and p-values estimated by Fischer exact tests are cumulative. All statistical analyses were performed using the SPSS/PASW (version 15.0.1 and 17) software package.

## Results

### SNP344: Haplotype status and ethnic distribution

**Figure 1:**
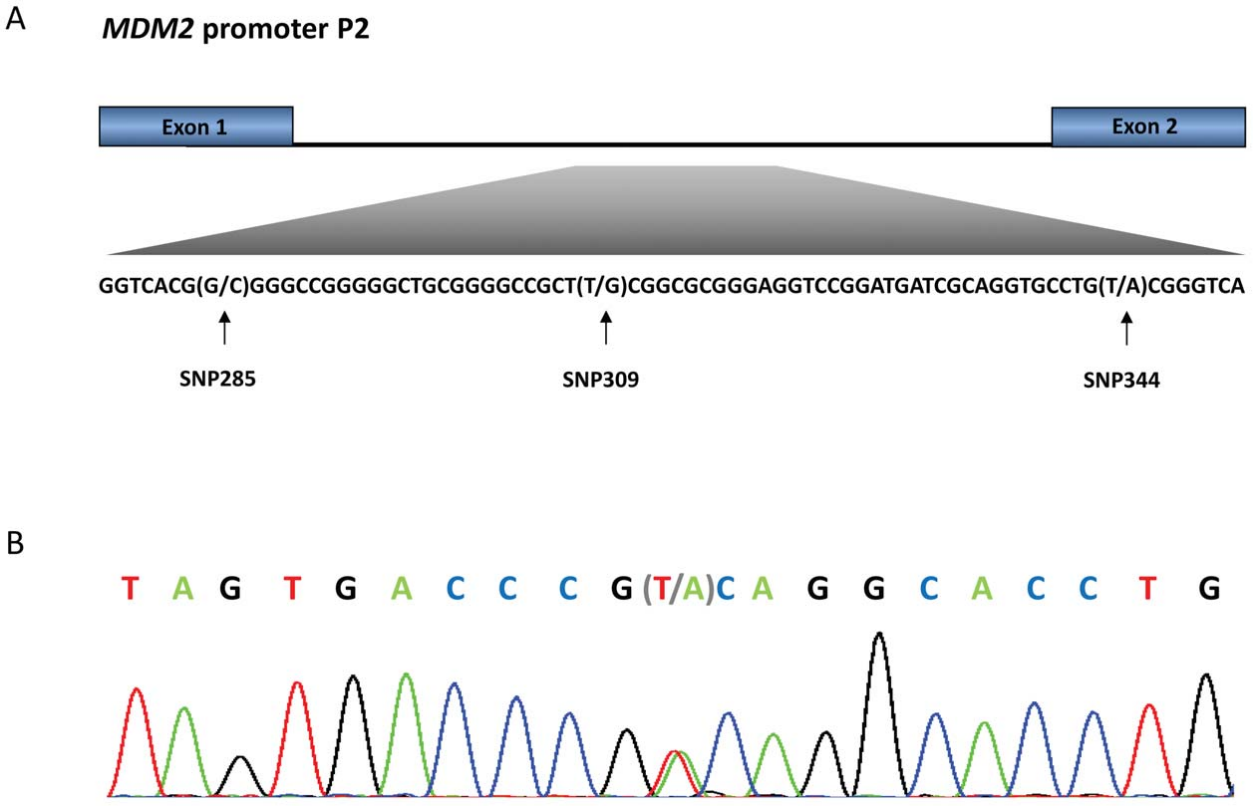
*MDM2* promoter P2. (**A**) The promoter is located between exon 1 and 2 of the *MDM2* gene and harbours SNP285 (rs117039649), SNP309 (rs2279744) and SNP344 (1196333). (**B**) Representative sequencing chromatogram from an individual heterozygous for SNP344 (sequence showed as reverse complementary to the sense strand).

SNP344 (rs1196333) is located within the *MDM2* promoter P2, 344 bps downstream of exon 1 (Figure 1). Among 2,954 healthy Norwegian controls, we observed the SNP344A-variant in 181 individuals (6.1%). One individual harboured the homozygous SNP344AA genotype, while 180 were heterozygous (SNP344TA; Table 1). Thus, the minor allele frequency was 3.1%, and the distribution of genotypes was in accordance with Hardy-Weinberg equilibrium.

**Table 1:** Distribution of SNP344 genotypes in cancer patients and healthy controls.

	SNP344 (rs1196333) status			
Cohort	Genotype TT	Genotype TA	Genotype AA	Total
	*n* (%)	*n* (%)	*n* (%)	*n* (%)
Ovarian cancer	1831 **(95.0)**	95 **(4.9)**	1 **(0.1)**	1927 **(100)**
Breast cancer	1205 **(94.8)**	63 **(5.0)**	3 **(0.2)**	1271 **(100)**
Endometrial cancer	845 **(94.4)**	48 **(5.4)**	2 **(0.2)**	895 **(100)**
Prostatic cancer	594 **(92.7)**	46 **(7.2)**	1 **(0.2)**	641 **(100)**
Healthy controls	2773 **(93.9)**	180 **(6.1)**	1 **(0.0)**	2954 **(100)**

Notably, we observed the SNP344A-variant only among individuals harbouring the SNP309TT or TG genotype, strongly indicating SNP344A to be located on the SNP309T-allele, making a distinct SNP309T/344A haplotype (p<1×10^−10^). Further, since SNP285C is located on the SNP309G-allele, one may deduce that SNP344A only exist in the SNP285G/309T/344A haplotype.

In a cohort of African Americans (n = 50) we found 17 (34%) individuals to harbour SNP344A (one homozygous and 16 heterozygous). This frequency was significantly higher as compared to the frequency observed among Caucasians (p<0.001). Notably, the distribution of SNP344 among African Americans was in line with the limited data on this SNP presented in the Ensembl database. As for Caucasians, we found the SNP344A-allele only among African Americans harbouring the SNP309T-allele.

### Effect of SNP344 status on transcription factor binding

In order to evaluate the potential impact of SNP344 status on transcription factor binding, we performed *in silico* analyses using the JASPAR database [15], predicting transcription factor binding to the SNP344T and A-alleles. Using the “wild-type” SNP344T-allele sequence and a profile score threshold of 80% (default settings) in the database search, two transcription factor binding sites were identified including the position of SNP344: one binding site for TFAP2A and one for SPIB (Table 2). When substituting the SNP344T with the A-variant, the predicted binding strength of TFAP2A was slightly increased while the site for SPIB was disrupted. In addition, introduction of the A generated a novel binding site for AP1. Thus, transcription efficacy from the A-allele, as compared to the T-allele, could either be reduced due to a disrupted SPIB site or increased due to enhanced binding of TFAP2A and a novel AP1 site.

**Table 2:** Effect of SNP344 status on transcription factor binding to MDM2 promoter P2.

Transciption factor	Binding score	
	SNP344T	SNP344A
TFAP2A	6.65	8.03
SPIB	4.68	-
AP1	-	6.20

### SNP344 status and MDM2 expression levels

**Figure 2:**
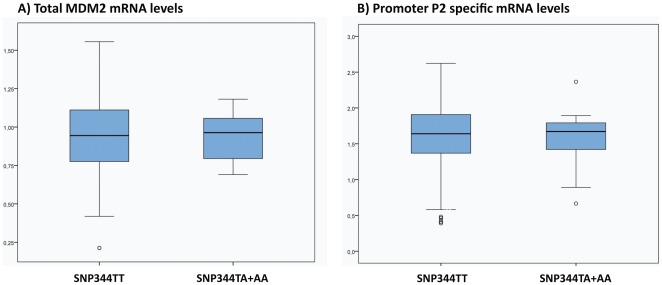
SNP344 and mdm2 expression. Box-plots representing log transformed relative levels of total MDM2 mRNA (**A**) and promoter P2 specific mRNA (**B**) in individuals harbouring the SNP344TT genotype versus the TA and AA genotypes.

To assess the potential impact of SNP344 genotype on MDM2 expression, we analysed MDM2 mRNA levels in leukocytes from a subgroup of 215 healthy young males by qPCR. No difference in MDM2 expression level between individuals harbouring the SNP344AA (n = 1), 344TA (n = 10) or 344TT (n = 204) genotypes was recorded (p>0.5 Figure 2A).

Since the SNP344A-variant resides on the SNP309T allele, we performed separate sub-group analyses restricted to individuals harbouring the SNP309TG (n = 101) or 309TT (n = 75) genotype. No difference in MDM2 expression level related to SNP344 status was recorded in any of these subgroups (p>0.4)

Since one may assume that the SNP344 status only affects the MDM2 expression from promoter P2, in which the SNP is localised, and that the effect of this SNP may be masked in assays analysing the total MDM2 expression levels, we performed similar qPCR experiments as described above but specific for mRNA originating from promoter P2. No association between SNP344 status and promoter P2 specific expression was observed (p>0.5).

### SNP344 status and cancer risk

In order to evaluate the potential impact of SNP344 status on cancer risk, we compared the frequency of SNP344 variants among ovarian (n = 1,927), breast (n = 1,271), endometrial (n = 895) and prostate cancer patients (n = 641) to healthy controls (n = 2,954). The results are summarised in Table 1. We found no significant differences between the frequency of SNP344A in any of the analysed cancer groups and the healthy controls.

**Figure 3:**
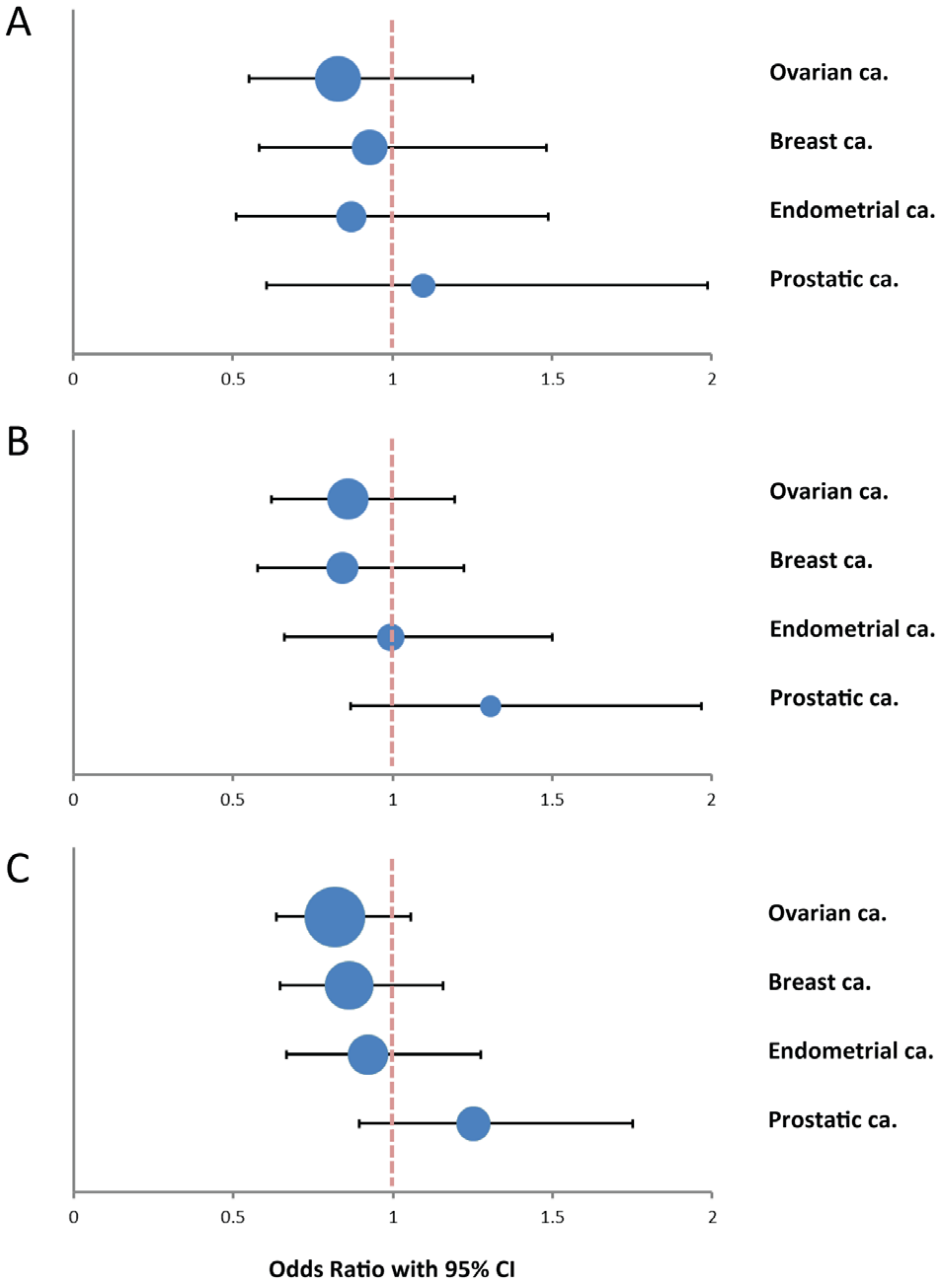
Impact of SNP344A on cancer risk. Forrest plot showing the effect of SNP344A on risk of ovarian, breast, endometrial and prostatic cancer, as compared to healthy controls, among individuals harbouring the SNP309TG genotype (A), the SNP309TT genotype (B) and the TG and TT genotypes combined (C).

Given that SNP344A was linked to the SNP309T-allele, as described above, individuals harbouring the SNP309GG genotype may be censored as non-informative with respect to the effect of SNP344. We therefore assessed the impact of SNP344 on cancer risk among individuals harbouring SNP309T-allele only (SNP309TG or TT genotype; Table 3). We did not find any association between SNP344 status and any of the cancer forms analysing the SNP309TG and 309TT carriers separately or combined (Figure 3).

**Table 3:** Distribution of SNP344 genotypes in cancer patients and healthy controls, restricted to individuals carrying the SNP309T-allele.

	SNP344 (rs1196333) status			
Cohort	Genotype TT	Genotype TA	Genotype AA	Total
	*n* (%)	*n* (%)	*n* (%)	*n* (%)
Ovarian cancer	1568 **(94.2)**	95 **(5.7)**	1 **(0.1)**	1664 **(100)**
Breast cancer	1022 **(93.9)**	63 **(5.8)**	3 **(0.3)**	1088 **(100)**
Endometrial cancer	725 **(93.5)**	48 **(6.2)**	2 **(0.3)**	775 **(100)**
Prostatic cancer	502 **(91.4)**	46 **(8.4)**	1 **(0.2)**	549 **(100)**
Healthy controls	2421 **(93.0)**	180 **(6.9)**	1 **(0.0)**	2602 **(100)**

### SNP344 status and clinical parameters

We further assessed the potential impact of SNP344 status on several clinical parameters among the patients included in the case-control comparisons described above.

Data for age of onset of disease was available for all endometrial (n = 895) and prostate cancer patients (n = 641) as well as large sub-cohorts of breast (n = 1,173) and ovarian cancer patients (n = 761). We found no effect of SNP344-status on age of onset in either of the four cancer forms when comparing total patient groups for each cancer form or subgroups stratified according to SNP309-status (all p-values>0.15).

Among the breast cancer patients analysed, many were enrolled in prospective studies aiming at identifying genetic mechanisms of resistance to chemotherapy; n = 106 from two studies evaluating either doxorubicin monotherapy or a combined 5-fluorouracil/mitomycin regimens [16], [17], while n = 201 were obtained from a study randomizing between epirubicin and paclitaxel monotherapy [18], [19]. Thus, for these patients we had detailed records for objective response to therapy in the neoadjuvant setting. SNP344 status did not affect response to either DNA damaging drugs (doxorubicin, mitomycin) or spindle poison (paclitaxel; p>0.1 for all comparisons) in these studies, although the conclusion here may be uncertain due to limited numbers of SNP344A-alleles observed. The potential effect of SNP344 status on relapse-free or overall survival could be assessed in these studies due to the limited number of individuals harbouring the 344A-allele.

In a previous study, we found MDM2 SNP285 status to correlate to stage in endometrial carcinomas [14]. Here, no correlation between FIGO stage and SNP344 status was recorded. (all p-values>0.3).

## Discussion

MDM2 is an important factor regulating cellular homeostasis through its close interactions with proteins like p53, pRB and E2F1. Thus, MDM2 controls processes like growth arrest, apoptosis and senescence, and MDM2 gene amplification and enhanced translation have been observed in many tumour forms [2], [4], [5], [6], [7].

The importance of MDM2 expression in preventing cancer development is further underlined by the finding that the *MDM2* promoter P2 SNPs 285 and 309 both modulate transcription factor binding and affect the risk of multiple cancer forms [8], [11], [13], [14]. While the exact mechanism of transcription initiation from the *MDM2* promoter P2 is not known, this promoter is activated in response to cellular stress, and in addition to Sp1, P2 harbours binding sites for multiple transcription factors including p53, the estrogen receptor, AP1 [8], [14] as well as several others (prediction by the JASPAR database; data not shown).

SNP344T>A is the third *MDM2* promotor P2 polymorphism. Contrasting SNP285C, which is located on the SNP309G allele, SNP344A resides on the SNP309T allele. Here, we performed *in silico* prediction evaluating transcription factor binding strength and determined the effect of SNP344 status on MDM2 transcript levels in lymphocytes. While SNP344 was found to affect binding of the transcription factors TFAP2A, SPIB and AP1, no effect of SNP344 status on MDM2 transcription was recorded. Importantly, assessing the distribution of a SNP344 in a large cohort of healthy individuals and among patients suffering from ovarian, breast, endometrial and prostate cancer, we detected no differences with respect to SNP344 distribution between healthy individuals and cancer patients. While our study included a limited number of cancer forms, for three of these cancers (breast, ovary end endometrium) the SNP285C variant has previously been shown to affect individual risk in the same ethnic population [13], [14]. Thus, these malignancies represent suitable cancer forms to detect any potential effects of SNP344 status on disease risk.

Contrasting the SNP285G>C polymorphism which is detected among Caucasians only [13], SNP344A, similar to SNP309G, seems to be an ancient polymorphism that is also present among Africans. Interestingly, the distribution of the SNP309G variant allele, but also SNP344A, seems to vary across different ethnic groups. While the frequency of the SNP309G allele varies from ∼10% in Africans to ∼40% in Caucasians and ∼50% in Asians [12], the frequency of the SNP344A allele is about 18% in Africans but 3% only in Caucasians. This difference in ethnic distribution, taken together with the rapid spread of the young SNP285C polymorphism among Caucasians [20], indicates that all three *MDM2* promoter P2 polymorphisms may be subject to evolutionary selection under different living conditions. Thus, further investigations elucidating potential impact of SNP344 on biological function other than the cancer forms reported here may be warranted.
